# Members of the Cyr61/CTGF/NOV Protein Family: Emerging Players in Hepatic Progenitor Cell Activation and Intrahepatic Cholangiocarcinoma

**DOI:** 10.1155/2016/2313850

**Published:** 2016-10-18

**Authors:** Qunfeng Wu, Marda Jorgensen, Joanna Song, Junmei Zhou, Chen Liu, Liya Pi

**Affiliations:** ^1^Department of Pathology and Laboratory Medicine, Rutgers, The State University of New Jersey, Newark, NJ 07103, USA; ^2^Department of Pediatrics, University of Florida, Gainesville, FL 32610, USA

## Abstract

Hepatic stem/progenitor cells (HPC) reside quiescently in normal biliary trees and are activated in the form of ductular reactions during severe liver damage when the replicative ability of hepatocytes is inhibited. HPC niches are full of profibrotic stimuli favoring scarring and hepatocarcinogenesis. The Cyr61/CTGF/NOV (CCN) protein family consists of six members, CCN1/CYR61, CCN2/CTGF, CCN3/NOV, CCN4/WISP1, CCN5/WISP2, and CCN6/WISP3, which function as extracellular signaling modulators to mediate cell-matrix interaction during angiogenesis, wound healing, fibrosis, and tumorigenesis. This study investigated expression patterns of CCN proteins in HPC and cholangiocarcinoma (CCA). Mouse HPC were induced by the biliary toxin 3,5-diethoxycarbonyl-1,4-dihydrocollidine (DDC). Differential expression patterns of CCN proteins were found in HPC from DDC damaged mice and in human CCA tumors. In addition, we utilized reporter mice that carried* Ccn2/Ctgf* promoter driven GFP and detected strong* Ccn2/Ctgf* expression in epithelial cell adhesion molecule (EpCAM)^+^ HPC under normal conditions and in DDC-induced liver damage. Abundant CCN2/CTGF protein was also found in cytokeratin 19 (CK19)^+^ human HPC that were surrounded by *α*-smooth muscle actin (*α*-SMA)^+^ myofibroblast cells in intrahepatic CCA tumors. These results suggest that CCN proteins, particularly CCN2/CTGF, function in HPC activation and CCA development.

## 1. Introduction

The normal liver fosters a small subset of adult stem cells that reside quiescently in the Canals of Hering, which is a transitional zone located between the bile canaliculi and interlobular ductal systems [1]. In response to massive parenchymal damage or severe impairment, if hepatocyte proliferation is arrested, the stem cells become activated in the form of ductular reactions and produce atypical ductular cells, also termed hepatic progenitor cells (HPC) in humans or oval cells in rodents [2, 3]. These cells are capable of aiding in both hepatocytic regeneration and biliary regeneration. HPC are heterogeneous populations that expand extensively around periportal areas and express certain biliary markers including epithelial cell adhesion molecule (EpCAM) and cytokeratin 19 (CK19) [4, 5]. The expansion of the HPC population represents an attempt to participate in the regeneration of damaged liver during chronic liver injury. However, persistent injury and the chronic inflammatory milieu activate profibrogenic signaling resulting in myofibroblast activation and collagen deposition. HPC activation is strongly correlated with fibrosis progression in many human chronic liver diseases including alcoholic and nonalcoholic steatohepatitis, chronic hepatitis C, cholestatic hepatitis, and hereditary haemochromatosis [6–11]. Moreover, HPC within the biliary tree have been gaining relevance in relation to liver cancer, particularly intrahepatic cholangiocarcinoma (CCA) and hepatocellular carcinoma (HCC). During chronic hepatitis B or C viral infection with accompanying liver cirrhosis, HPC proliferation is directly related to disease severity, suggesting that activation of the HPC compartment is associated with an increased risk of liver cancer development [7]. Clinical and experimental animal studies have demonstrated that progenitor cells can play a critical role in hepatic carcinogenesis and recruitment of progenitor cells contributing to liver cancer leading to HCC and intrahepatic CCA [12–15]. In addition, clinical-pathological correlation and risk factors for HCC and intrahepatic CCA support the role of HPC in different subtypes of liver cancers. Recent evidences indicate that HPC may be the cell of origin in certain subtypes of CCA such as cholangiolocarcinoma and the so-called mixed-type CCA or HCC tumors, such as CK19^+^ HCC [13, 16–18]. Lastly, combined hepatocellular cholangiocarcinoma (CHC), a rare form of primary liver cancer that accounts for 0.4%–14.2% of liver carcinomas [19], exhibits pathological features of HCC and CCA. It has been proposed that CHC directly derives from HPC activation and differentiation [20].

Matricellular proteins of the CCN (CYR61/CTGF/NOV) family are important signal modifiers in stem cell niches and can modulate multiple signaling pathways mediated by Notch, Wnt, and transforming growth factor-*β* (TGF-*β*) [3, 21, 22]. The CCN family is composed of six structurally conserved secreted proteins: CCN1/cysteine-rich 61 (CYR61), CCN2/connective tissue growth factor (CTGF), CCN3/nephroblastoma overexpressed (NOV), CCN4/Wnt1 induced secreted protein-1 (WISP-1), CCN5/WISP-2, and CCN6/WISP-3. All CCN proteins share a conserved four-modular structure with the exception of CCN5/WISP-2 which lacks a cysteine knot domain module. CCN proteins exert activity on various types of cells through their broad binding capabilities to ECM proteins, growth factors, and cell surface proteins [21]. CCN proteins modulate cell-matrix interaction and mediate cell adhesion, migration, differentiation, apoptosis, and survival. Altered levels of CCN expression or activities contribute to the pathogenesis of many diseases that occur when inflammation or tissue injury becomes chronic, such as liver fibrosis and liver cancer [21, 23–25]. In order to understand CCN function in HPC activation and CCA development, we examined the expression of CCN proteins in mouse HPC induced by 3,5-diethoxycarbonyl-1,4-dihydrocollidine (DDC), a biliary toxin that induces ductular reaction and biliary fibrosis [26]. The levels of CCN mRNAs were also quantitatively measured from human intrahepatic CAA. Within the family, CCN2/CTGF exhibited predominant expression in both mouse HPC and human CCA tumors.

## 2. Materials and Methods

### 2.1. Human Tissues

Human liver tissues were obtained through Shands Hospital according to an approved protocol approved by the institutional review board at the University of Florida. Written informed consents were obtained from all subjects. Tumor or adjacent nontumor tissues from intrahepatic CCA containing livers were separated after dissection and snap-frozen for molecular analyses.

### 2.2. The DDC Mouse Model

Transgenic mice carrying* Ccn2/Ctgf* promoter driven GFP* (Ctgfp-GFP)* were previously described [27]. Wild-type or* Ctgfp-GFP* mice at 8–10 weeks of age were fed with a diet supplemented with 0.1% DDC (Bio-Serv, Frenchtown, NJ) to induce cholangitis, ductular reactions, and biliary fibrosis. All protocols and procedures were approved by the University of Florida IACUC and were in accordance with National Institutes of Health guidelines.

### 2.3. Immunohistochemistry

All mouse liver tissues were fixed overnight in 4% paraformaldehyde to preserve the GFP fluorescence signal. Tissues were infiltrated with 20% sucrose before being embedded in OCT. 6 *μ*m sections were used in this study. GFP staining in combination with EpCAM antibody (eBioscience, San Diego, CA) or hepatocyte nuclear factor 4*α* (HNF4*α*) antibody (Santa Cruz Biotechnologies, Dallas, Texas) required antigen retrieval at 37°C for 10 minutes in trypsin (Digest-All 2, Invitrogen, Carlsbad, CA). Primary antibodies employed were 1 : 100 rat anti-EpCAM, 1 : 100 rabbit anti-HNF4*α*, and 1 : 750 chicken anti-GFP (Aves Labs, Tigard, OR). Alexa Fluor 488 conjugated goat anti-chicken and Alexa Fluor 594 conjugated donkey anti-rabbit secondary antibodies (Invitrogen, Carlsbad, CA) were used at 1 : 500 for detection. Human intrahepatic CCA and HCC tissue arrays were purchased from US Biomax, Inc. (Rockville, MD). Gomori's Trichrome staining for collagen fibers was carried out using a Thermo Scientific Chromaview collagen blue kit according to the manufacturer's instructions (Richard-Allan Scientific, Kalamazoo, MI). To evaluate fibrosis in DDC treated mice, liver sections were stained with Picrosirius Red solution (American MasterTech, Lodi, CA). In addition, staining for CK19, CCN2/CTGF, and *α*-SMA requires antigen retrieval in Trilogy pretreatment solution (Cell Marque, Rocklin, CA) at 95°C for 25 minutes. The sections were blocked in 3% horse serum (Vector Laboratories) for one hour prior to overnight incubation with anti-CK19 antibody (Abcam), anti-CCN2/CTGF antibody (Santa Cruz Biotechnologies), and anti-*α*SMA antibody (Sigma, St. Louis, MO). Additional IHC for CCN2/CTGF in liver sections of human intrahepatic CCA was carried out using a rabbit antibody (1 : 200, Abcam) and detected using a VECTASTAIN ABC-AP kit and Vector Red substrate (Vector Laboratories, Burlingame, CA).

### 2.4. RNA Isolation and Reverse Transcriptase-Polymerase Chain Reaction (RT-PCR) Analysis

Total RNAs were extracted using RNeasy Mini kit (Qiagen, Valencia, CA) followed by incubation with RQ1 RNase-free DNase (Promega, Madison, WI) to remove any genomic DNA contamination. 2 *μ*g of total RNA and 50 pmol random hexamer were used for Superscript™ III First-Strand cDNA Synthesis (Invitrogen). RT-PCR was carried out using 0.5 *μ*L cDNAs as templates, 0.2 *μ*M of each set of primers, and REDExtract-N-Amp™ tissue PCR mixtures (Sigma). Amplification conditions consisted of thirty cycles of denaturation at 94°C for 30 sec, annealing at 55°C for 30 sec, and extension at 72°C for 30 sec. Primer pairs for mouse genes include the following: 5′-TTTACAGTTGGGCTGGAAGC-3′ and 5′-CACCGCTCTGAAAGGGATCT-3′ for* Ccn1/Cyr61*, 5′-GTCTTCACACTGGTGCAGCC-3′ and 5′-ACTGGAAGACACATTTGGCC-3′ for* Ccn2/Ctgf*, 5′-CTTGGTGCGGAGACACTTTT-3′ and 5′-CGCCAGTGTGAGATGGTAAA-3′ for* Ccn3/Nov*, 5′-TGAGTGCTTGAGCATCCAGA-3′ and 5′-AATGAGAAAGGAGAGAGGCTGT-3′ for* Ccn4/Wisp1v*, 5′-GTGTCCAAGGACAGGCACAG-3′ and 5′-GCAACCCACTGATCCATCTT-3′ for* Ccn5/Wisp2*, 5′-AATGAGAAAGGAGAGGAGGCTGT-3′ and 5′-TGAGTGCTTGAGCATCCAGA-3′ for* Ccn6/Wisp3*, 5′-CCACCACGCTCTTCTGTCTAC-3′ and 5′-AGGGTCTGGGCCATAGAACT-3′ for* tumor necrosis factor (TNFα)*, 5′-CCACCGCAAATGCTTCTAAGT-3′ and 5′-GGCAGGAATGATTTGGAAAGG-3′ for *α-SMA*, 5′-TGTGTTCCCTACTCAGCCGTCT-3′ and 5′-CATCGGTCATGCTCTCTCCAA-3′ for* procollagen type α1(I)*, and 5′-TTGACGGAAGGGCACCACCAG-3′ and 5′-GCACCACCACCCACGGAATCG-3′ for* 18S ribosomal RNA*.* Wisp1v* gene lacking sequence corresponding to Von Willbred Factor type C (VWC) domain is a variant of* Wisp1* gene. It was chosen in this study because of its association with cholangiocarcinoma [28].

In real-time RT-PCR analysis, cDNAs from CCA samples were analyzed in ABI Prism 7900 HT Fast Real-Time (Applied Biosystems, Carlsbad, California). Primer pairs for human genes used are as follows: 5′-TCACCCTTCTCCACTTGACC-3′ and 5′-AGTCCTCGTTGAGCTGCTTG-3′ for* CCN1/CYR61*, 5′-TGGAGATTTTGGGAGTACGG-3′ and 5′-CAGGCTAGAGAAGCAGAGCC-3′ for* CCN2/CTGF*, 5′-AAGAGCTGTGGTATGGGGTTC-3′ and 5′-GGTGGATGGCTTTGAGTGAC-3′ for* CCN3/NOV*, 5′-GTAAGATGTGCGCTCAGCAG-3′ and 5′-ACTGGGCGTTAACATTGGAG-3′ for* CCN4/WISP1v*, 5′-AGCCCAAGGACCCCAGTT-3′ and 5′-TCTCCAGTCGGCAGAAGC-3′ for* CCN5/WISP2*, 5′-CTGGCCTGGCACAGTTCT-3′ and 5′-TCTCTCACCAGGCTCACTCC-3′ for* CCN6/WISP3*, and 5′-GATGAGATTGGCATGGCTTT-3′ and 5′-GAGAAGTGGGGTGGCTT-3′ for *β*-*ACTIN*. The relative amount of target mRNA was calculated using the delta CT method and normalized against *β*-*ACTIN* as reference gene in each sample.

### 2.5. Statistical Analysis

Microsoft Excel software (Microsoft Corp., Redmond, WA) was used for statistical analysis. Data were represented as mean ± SD. Statistical significance (*P* < 0.05) was evaluated using Student's* t*-test and one-way analysis of variance (ANOVA). All experiments were performed a minimum of three times.

## 3. Results

### 3.1. Dynamic Expression of CCN Proteins during HPC Activation and Biliary Fibrosis Induced by DDC Damage in Mice

**Figure 1:**
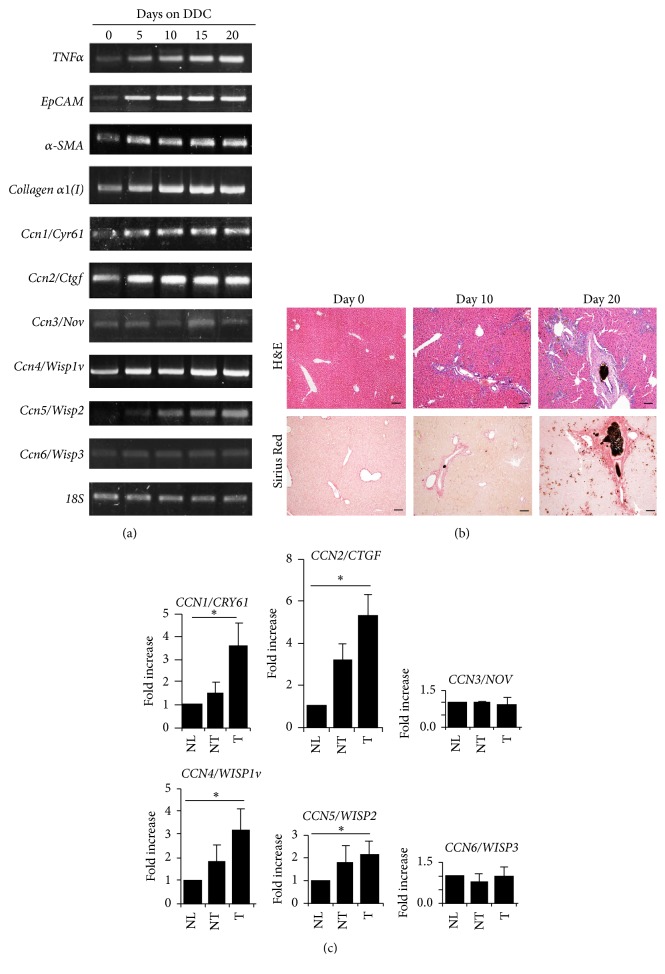
Dynamic expression of CCN proteins in HPC and human CCA tumors. (a) Transcriptional levels of the proinflammatory gene* TNFα*, the HPC marker* EpCAM*, and fibrosis related genes *α-SMA* and* collagen α1(I)* were measured by RT-PCR analysis to assess the development of HPC activation and biliary fibrosis in mice that were fed DDC for 0–20 days. CCN transcripts were determined in the DDC damaged mouse livers (a) or in human CCA tissues by quantitative RT-PCR analysis (c). (b) H&E staining and Sirius Red staining show the development of ductular reaction and liver fibrosis in DDC-fed mice. Scale bar: 250 *μ*m. (c) Data were calculated from CCA and corresponding nontumor samples from five human livers as compared to normal livers from three healthy donors. NL: normal liver from healthy donors; NT: adjacent nontumor tissues from CCA patients; T: tumor tissues from CCA patients. ^*∗*^ *P* < 0.05.

DDC is a porphyrinogenic agent widely used to study mechanisms of HPC/oval cell activation and proliferation [29]. DDC feeding is associated with increased biliary porphyrin secretion and segmental bile duct obstruction leading to pronounced pericholangitis, ductular reaction, activation of periductal myofibroblasts, and extensive collagen deposition in periportal areas within treated livers [26]. To monitor these changes, we fed wild-type C56BL6/J mice with DDC-containing diets for 0–20 days. Total RNA was extracted from liver homogenates. As shown in Figure 1(a), DDC-induced liver damage was verified in RT-PCR analysis based on upregulation of the proinflammatory gene* TNFα* at 5, 10, 15, and 20 days after treatment. Accordingly, ductular reactions occurred as early as day 5, indicated by induction of the HPC marker* EpCAM*. In addition, fibrosis related genes including *α-SMA* and* procollagen α1(I)* were upregulated in the DDC damaged livers, suggesting a concomitant fibrosis in response to DDC toxicity. The development of ductular reactions and liver fibrosis was confirmed in DDC-fed mice using both H&E staining and Sirius Red staining as shown in Figure 1(b). We also detected sustained induction of* Ccn1/Cyr61*,* Ccn2/Ctgf*, and* Ccn4/Wisp1v* mRNAs from 5 to 20 days after DDC feeding. In addition,* Ccn5/Wisp2* transcript was gradually increased in a temporal and spatial pattern similar to that of* TNFα* and reached a peak at day 20. By contrast,* Ccn3/Nov* and* Ccn6/Wisp3* did not show significant induction. These differential expression patterns suggested the involvement of* Ccn1/Cyr61*,* Ccn2/Ctgf*,* Ccn4/Wisp1v*, and* Ccn5/Wisp2* in DDC-induced liver injury.

### 3.2. Altered Expression of CCN Proteins in Intrahepatic CCA Tumors

CCN proteins are important regulators in stem cells and tumorigenesis. Expression of CCN family members has been shown to correlate with the clinical features of HCC [25]. To further determine whether CCN proteins were involved in liver cancer development, we extracted total RNA from intrahepatic CCA tumors as well as their adjacent normal counterparts. Expression patterns of CCN proteins in these tissues were compared with normal human liver tissues by RT-PCR analysis. Consistent with previous reports detailing overexpression of* CCN2/CTGF* and* CCN4/WISP1v* in CCA [27, 30], we detected significant overexpression of these two transcripts in all tested tumor tissues from our tumor samples (Figure 1(c)). In addition, induction of* CCN1/Cyr61* and* CCN5/WISP2* was also found in the CCA tumors, whereas* CCN3/NOV* and* CCN6/WISP3* did not have obvious changes in both the nontumor and tumor samples from the CCA tissues (Figure 1(c)). These results indicate that* CCN1/Cyr61*,* CCN2/CTGF*,* CCN4/WISP1v*, and* CCN5/WISP2* are involved in CCA tumorigenesis.

### 3.3. Specific Promoter Activity of the* Ccn2/Ctgf *Gene in Mouse HPC under Both Normal and DDC-Induced Liver Damage Conditions

**Figure 2:**
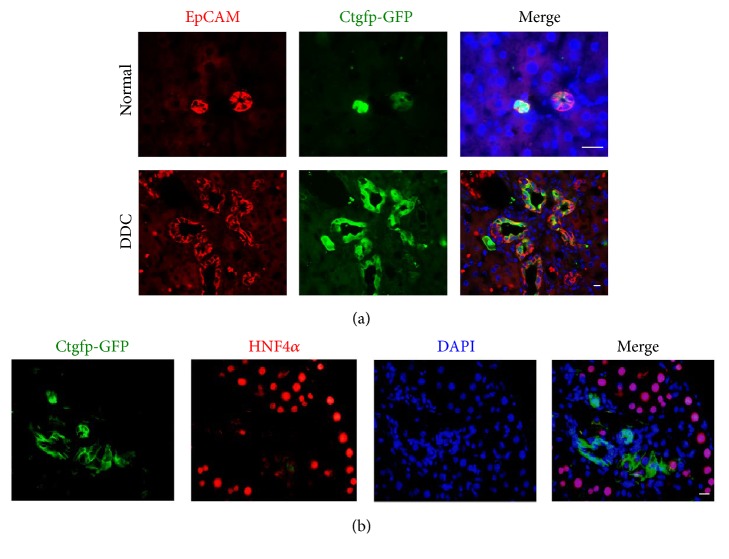
Immunofluorescent staining shows strong induction of* Ccn2/Ctgf* promoter activity in DDC-induced mouse HPC. (a) Dual staining for EpCAM and GFP proteins was carried out on liver sections from mice that carried* Ccn2/Ctgf* promoter driven GFP* (Ctgfp-GFP)* transgene under normal conditions or after 20-day DDC feeding. (b) Double staining for HNF4*α* and GFP showed little* Ccn2/Ctgf* promoter activity in mature hepatocytes of damaged livers from mice that were fed DDC for 20 days. Scale bar: 35 *μ*m.

The* CCN2/CTGF* gene had a very high level of induction in both DDC damaged mouse livers and CCA tumors as shown in Figures 1(a) and 1(c). To verify the expression of this gene in HPC, we took advantage of reporter mice carrying the* Ctgfp-GFP* transgene and visualized* Ccn2/Ctgf* expression* in vivo* based on GFP fluorescence. Mouse HPC were labeled using an antibody for the HPC marker EpCAM. As shown in Figure 2, confocal microscopy analysis detected specific* Ccn2/Ctgf* promoter activity in EpCAM^+^ HPC from subsets of small ducts in untreated mice. In response to DDC-induced liver damage, EpCAM^+^ HPC expanded in large numbers within the periportal zones. The majority of the EpCAM^+^ HPC were GFP positive, indicating predominant expression of the* Ccn2/Ctgf* gene in activated mouse HPC. By contrast, only vascular endothelial cells (EC) lining central veins exhibited detectable* Ccn2/Ctgf* promoter activity in DDC damaged livers (Supplemental Figure  1 in Supplementary Material available online at http://dx.doi.org/10.1155/2016/2313850). Little* Ccn2/Ctgf* promoter activity was found in HNF4*α* ^+^ hepatocytes located at periportal and pericentral areas during DDC-induced liver injury as shown in Figure 2(b) and Supplemental Figure  1. These observations were further supported by immunofluorescent staining for glutamine synthetase, an enzyme specifically expressed in hepatocytes around zone 3 of the liver acinus in the livers [31]. Lastly, absence of* Ccn2/Ctgf* promoter activity was found in mesenchymal cells that expressed vimentin (Supplemental Figure  3), implying that CCN2/CTGF was not directly involved in epithelial-mesenchymal transition during DDC-induced liver injury in mice.

### 3.4. Overexpression of CCN2/CTGF Protein in CK19^+^ HPC Surrounded by *α*-SMA^+^ Myofibroblast Cells in Fibrotic Human Intrahepatic CCA Tumors

**Figure 3:**
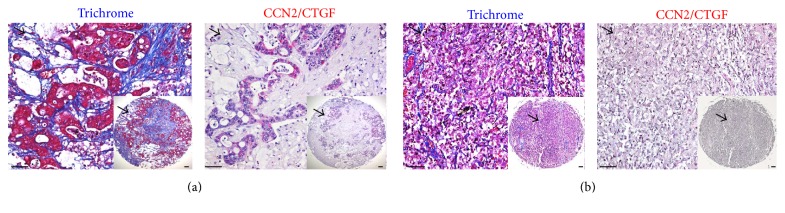
Immunofluorescent staining detects abundant CCN2/CTGF protein in fibrotic human CCA tumors. Trichrome or CCN2/CTGF staining was carried out on human intrahepatic CCA tissue arrays. Representative images for human CCA tumors (a) or non-CCA control tumors (pheochromocytoma) from adrenal glands (b) were shown. Inserts are macroscopic views of the stained tissues. Scale bar: 100 *μ*m. Arrowheads indicate the same location in each set of images in Figure 3.

**Figure 4:**
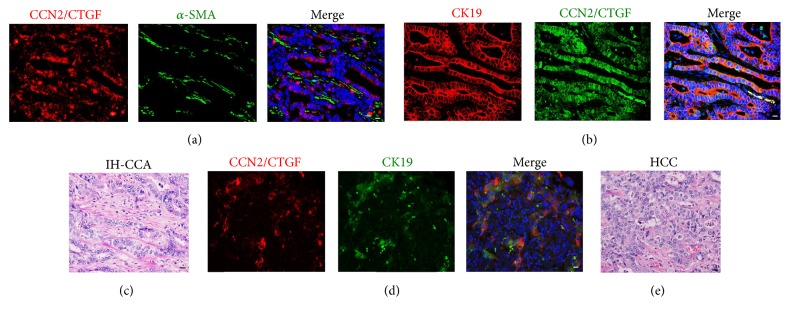
Localization of CCN2/CTGF protein in CK19^+^ human HPC that are surrounded by intratumoral *α*-SMA^+^ myofibroblast cells. Immunofluorescent staining for CCN2/CTGF in combination with *α*-SMA (a) or CK19 (b) was carried out on human CCA sections. (d) The immunofluorescent staining revealed CCN2/CTGF localization in CK19^+^ human HCC tumor cells. (c and e) IH-CCA and HCC tumors in (b) and (d) were histologically analyzed by H&E staining. Scale bar: 30 *μ*m.

The tumor microenvironment is a very important factor in the regulation of angiogenesis, invasion, and metastasis. We observed thick collagen fibrils in CCA stromal tissues that surrounded the tumor epithelial cells using Trichrome staining in Figure 3(a). Immunohistochemistry on consecutive sections showed abundant CCN2/CTGF localization in disorganized hepatic parenchyma characterization by extensive ductular reaction and mixed HPC populations in the IH-CCA (Figure 3(b)). This was in contrast to weak staining in pheochromocytoma from adrenal glands as a negative control. Additional immunofluorescent staining in Figure 4(a) suggested that *α*-SMA^+^ myofibroblast cells were not the main cellular source of CCN2/CTGF protein. CK19 is a marker that labels HPC, liver cancer stem cells, and ductular reactions in humans. Consistent with our recent finding [27], dual staining detected overexpression of CCN2/CTGF protein in CK19^+^ HPC characterized by atypical ductular cells without clearly defined lumens in IH-CCA tumors (Figures 4(b) and 4(c)). High level of CCN2/CTGF protein was also found in CK19^+^ HCC tumors (Figures 4(d) and 4(e)). Because CK19 positive HCC has been described as a cancer originating from HPC [14, 17], these results support a role for CCN2/CTGF in HPC activation during IH-CCA and HCC tumorigenesis.

## 4. Discussion

CCN family members are small, secreted cysteine-rich proteins modulating key pathways involved in initiation and resolution of normal wound healing and fibrosis after tissue damage. For example, CCN2/CTGF is overexpressed in many kinds of fibrotic disorders [32]. During liver damage, this protein is produced by a variety of different cell types including hepatic stellate cells, biliary epithelial cells, hepatocytes, and Kupffer cells [32]. It promotes fibrogenesis and survival in activated hepatic stellate cells through direct interaction with various subtypes of integrins leading to enhanced adhesive signaling [33].* In vivo* overexpression of CCN2/CTGF in hepatocytes does not result in hepatic injury or fibrosis per se but renders the livers more susceptible to the injurious actions of other fibrotic stimuli in mice [34]. Similarly, CCN4/WISP1 is profibrotic, since blockade of this molecule decreases experimental liver fibrosis* in vivo* [35, 36]. In contrast, CCN1/CYR61 and CCN3/NOV are inhibitors of the fibrotic response [37, 38]. CCN1/CYR61 induces senescence in hepatic myofibroblasts leading to attenuated TGF-*β* signaling, while its overexpression results in ER stress-related apoptosis in hepatic stellate cells [39]. Inhibition of CCN3/NOV using small interfering RNA enhances fibrotic gene expression in hepatic stellate cells [40]. Given the fact that liver fibrosis occurs as a final outcome of an abnormal wound healing response and is closely associated with HPC activation and ductular reactions during chronic liver disease, we speculate that the CCN proteins also regulate HPC activation. In supporting this notion, a recent report identifies CCN1/CYR61 as a critical regulator in biliary injury repair through the integrin *α*v*β*5/NF-*κ*B/JAG1 signaling axis [41]. CCN1/CYR61 stimulates* Jag1* expression in hepatic stellate cells and promotes HPC differentiation, cholangiocyte proliferation, and ductular reactions [41]. CCN2/CTGF is another member involved in HPC activation and ductular reactions. We have identified this molecule as one of several differentially expressed genes in oval cells from regenerating rat livers following 2-acetylaminofluorene and partial hepatectomy [42]. CCN2/CTGF binds to fibronectin and promotes rat oval cell adhesion and migration [43]. Moreover, CCN2/CTGF functions together with integrin *α*v*β*6 and enhances TGF-*β* activation. Conditional deletion of exon 4 of this gene reduces ductular reactions and biliary fibrosis [27]. In this study, we observed that* Ccn2/Ctgf* had a relatively higher level of induction than other CCN proteins in DDC damaged mouse livers in Figure 1(a). Specific promoter activity of the* Ccn2/Ctgf* gene was found in HPC from normal and DDC damaged mouse livers (Figure 3), suggesting an important role for* Ccn2/Ctgf* in HPC activation and ductular reactions. On the other hand, we observed upregulation of* Ccn4/Wisp1v* and* Ccn5/Wisp2* in DDC damaged mouse livers as shown in Figure 1(a). Thus,* Ccn4/Wisp1v* and* Ccn5/Wisp2* are potential mediators for HPC activation and biliary fibrosis, although their cellular sources and functions need to be defined in future studies.

CCN proteins are emerging as a unique group of extracellular signaling modulators involved in establishment, growth, and metastases in liver cancer via interaction of cancer cells with the intratumor stroma [24]. The majority of CCN family members are induced by growth factors, cytokines, and cellular stress such as inflammation. Altered expressions of CCN1/CYR61, CCN2/CTGF, CCN3/NOV, and CCN4/WISP1 were found to correlate with clinical features, including venous invasion, cellular differentiation, TNM staging for malignant tumors, disease-free survival, and overall survival in HCC [25]. Both positive and negative associations of CCN1/CYR61 with HCC have been reported [25, 44, 45]. CCN2/CTGF and CCN3/NOV have been shown to promote HCC development [46, 47]. CCN4/WISP1 is negatively linked with HCC, whereas CCN5/WISP2 effects no significant change in HCC development [25]. In the CCA tumor setting, only CCN2/CTGF and CCN4/WISP1v have been studied. CCN2/CTGF has been identified as an independent prognostic indicator of both tumor recurrence and overall survival for intrahepatic CCA patients regardless of tumor location, tumor grade, or vascular and perineural invasion [30]. CCN4/WISP1v can stimulate the invasive phenotype of CCA cells with activation of both p38 and p42/p44 mitogen-activated protein kinases [28]. Overexpression of CCN4/WISP1v is associated with lymphatic and perineural invasion of CCA tumor cells and a poor clinical prognosis [28]. This study showed significant upregulation of CCN2/CTGF and CCN4/WISP1v mRNAs in our CCA tumors (Figure 1(c)). Immunohistochemistry demonstrated localization of CCN2/CTGF in HPC and cholangiocytes within the CCA tissues (Figures 3 and 4). In addition, we observed the induction of CCN1/CYR61 and CCN5/WISP2 but no significant change of CCN3/NOV and CCN6/WISP3 in CCA (Figure 1(c)). Based on these dynamic expression patterns, it is consistent to postulate that, like CCN2/CTGF and CCN4/WISP1v, CCN1/CYR61 and CCN5/WISP2 are critical regulators in CCA development. Characterization of cell types expressing CCN1/CYR61 and CCN5/WISP2 and investigation of exact action mediated by the two molecules would be interesting directions to address the molecular mechanism of CCA tumorigenesis in the future.

In summary, cell-cell and cell-matrix interactions are crucial for stem cell/progenitor function and liver cancer progression. In the HPC niche, myofibroblast cells, immune cells, cytokines, matricellular proteins, and inflammatory proteins are orchestrated along with specialized ECM to promote progenitor cell differentiation and CCA development. The differential spatiotemporal expression profiles among CCN proteins indicate that they might be required at precise time slots during these pathological processes. CCN proteins might act alone or in concert to exert overlapped or antagonistic functions. Genetic approaches are a powerful tool in determining protein function. Since germline, conditional, and tissue specific knockout mice for several CCN members are available [27, 41, 48–52], it is anticipated that utilization of these genetic materials will help address the detailed mechanisms involved in CCN-mediated regulation in the future. And these studies will provide further insight into HPC activation, ductular reactions, liver fibrosis, and CCA progression during chronic liver disease.

## 5. Conclusion

We report differential expression patterns of CCN proteins in HPC from DDC damaged mouse livers and human CCA tumors. Strong induction and specific localization indicate the importance of the profibrotic CCN2/CTGF member in HPC activation and CCA development.

## Supplementary Material

Supplemental materials and methods Ctgfp-GFP mice (eight-week old) were fed a 0.1% DDC-supplemented diet for 0 or 20 days before being sacrificed to determine the expression pattern of Ccn2/Ctgf promoter driven GFP in vivo. Liver slices were fixed in 4% paraformaldehyde. OCT embedding was performed for vimentin staining and paraffin embedding was for HNF4α and glutamine synthetase. In the immunofluorescent staining, 10% horse serum was used to block nonspecific signals. Primary antibodies included chicken anti-GFP, rabbit anti-HNF4α, mouse anti-glutamine synthetase (Abcam, Cambridge, MA), and chicken anti-vimentin (EnCor Biotechnology, Gainesville, FL). Alexa Fluor 488 conjugated donkey anti-goat and Alexa Fluor 594 conjugated donkey anti-rabbit secondary antibodies (Invitrogen, Carlsbad, CA) were used for detection. Images were taken under fluorescent microscope using DP80 color camera and cellSens software (Olympus, Pittsburgh, PA).

## Abbreviations

CCN: Cyr61/CTGF/NOV
Cyr61: Cysteine-rich angiogenic inducer 61 protein
CTGF: Connective tissue growth factor
NOV: Nephroblastoma overexpressed protein
WISP1v: WNT1 inducible signaling pathway protein 1 variant
WISP2: WNT1 inducible signaling pathway protein 2
WISP3: WNT1 inducible signaling pathway protein 3
CCA: Cholangiocarcinoma
HCC: Hepatocellular carcinoma
HPC: Hepatic progenitor cell
CHC: Combined hepatocellular cholangiocarcinoma
EpCAM: Epithelial cell adhesion molecule
CK19: Cytokeratin 19.
