# Supplementary figures and images for: Members of the Cyr61/CTGF/NOV Protein Family: Emerging Players in Hepatic Progenitor Cell Activation and Intrahepatic Cholangiocarcinoma
Source: Gastroenterol Res Pract. 2016 Oct 18;2016:2313850. doi: 10.1155/2016/2313850 (PMC5088274; doi:10.1155/2016/2313850)

Supplemental Figure 1

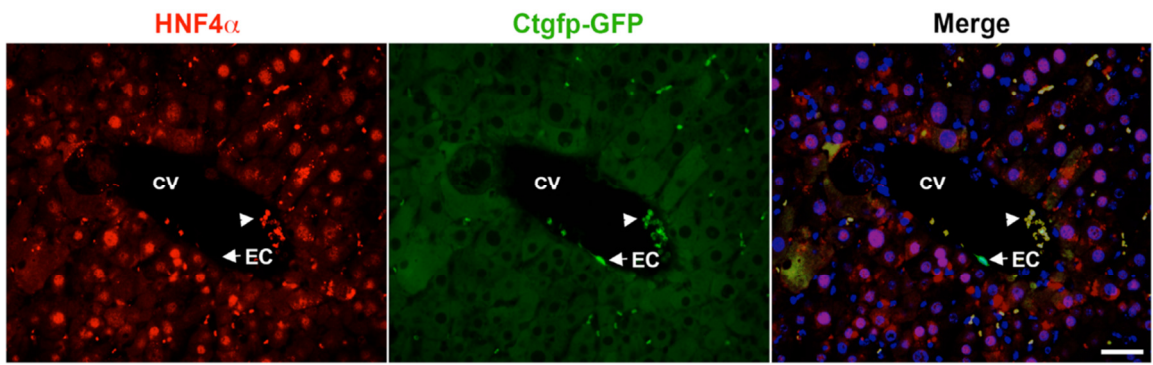

Supplemental Figure 2

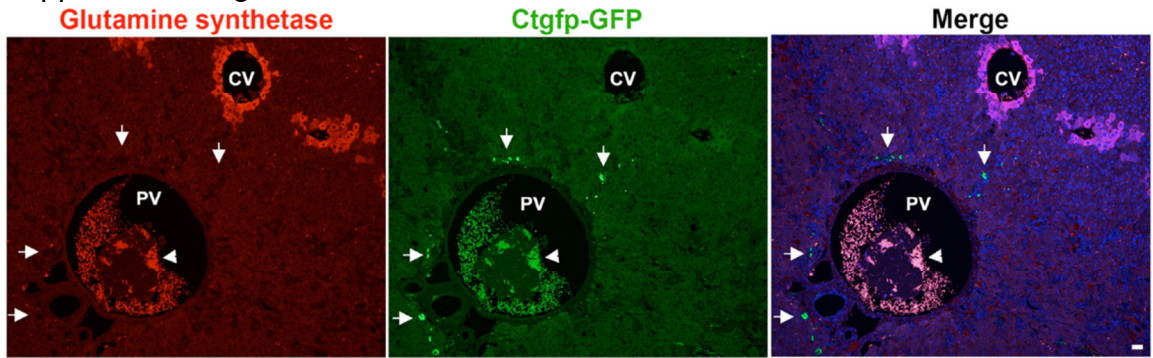

Supplemental Figure 3

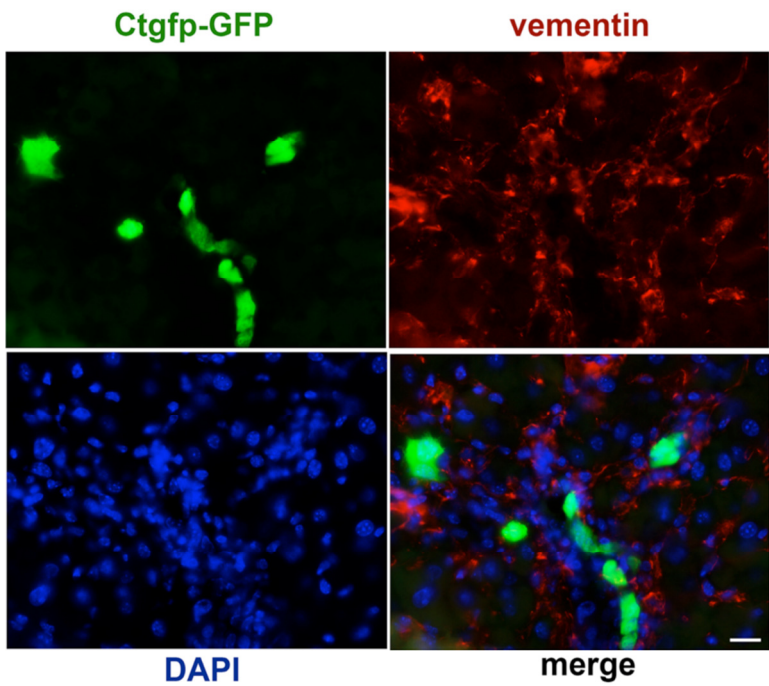

Supplement: Supplementary file 1 — Supplemental materials and methods Ctgfp-GFP mice (eight-week old) were fed a 0.1% DDC-supplemented diet for 0 or 20 days before being sacrificed to determine the expression pattern of Ccn2/Ctgf promoter driven GFP in vivo. Liver slices were fixed in 4% paraformaldehyde. OCT embedding was performed for vimentin staining and paraffin embedding was for HNF4α and glutamine synthetase. In the immunofluorescent staining, 10% horse serum was used to block nonspecific signals. Primary antibodies included chicken anti-GFP, rabbit anti-HNF4α, mouse anti-glutamine synthetase (Abcam, Cambridge, MA), and chicken anti-vimentin (EnCor Biotechnology, Gainesville, FL). Alexa Fluor 488 conjugated donkey anti-goat and Alexa Fluor 594 conjugated donkey anti-rabbit secondary antibodies (Invitrogen, Carlsbad, CA) were used for detection. Images were taken under fluorescent microscope using DP80 color camera and cellSens software (Olympus, Pittsburgh, PA). [file 2313850.f1.pdf]
